# Seroepidemiology of human enterovirus71 and coxsackievirusA16 among children in Guangdong province, China

**DOI:** 10.1186/1471-2334-13-322

**Published:** 2013-07-15

**Authors:** Wei Li, Lina Yi, Juan Su, Jing Lu, Hanri Zeng, Dawei Guan, Cong Ma, Wanly Zhang, Hong xiao, Hui Li, Yonghui Zhang, Jinyan Lin, Changwen Ke

**Affiliations:** 1 Department of Pathogen Detection Research for Emerging Infectious Diseases, Center for Disease Control and Prevention of Guangdong Province, Guangzhou, China; 2 Key Laboratories of Pathogen Detection for Emergency Response, Center for Disease Control and Prevention of Guangdong Province, Guangzhou, Guangdong, China; 3 Key Laboratories of Depository and Application for Pathogenic Microbiology, Center for Disease Control and Prevention of Guangdong Province, Guangzhou, Guangdong, China; 4 Guangdong Provincial Institution of Public Health, Guangzhou, Guangdong, China

**Keywords:** Enterovirus 71, Coxsackievirus A 16, Seroprevalence

## Abstract

**Background:**

Hand, foot and mouth disease (HFMD) is a common pediatric illness. Mainly induced by the Enterovirus 71 and Coxsackievirus A 16 infections, the frequently occurred HFMD outbreaks have become a serious public health problem in Southeast Asia. Currently,only a few studies have investigated the human immunity to HFMD in China. In this study, we conducted a cohort study in Guangdong province, China.

**Methods:**

Stored serum samples from children less than 10 years old were analyzed. The levels of EV71 and CA16 specific antibodies before, during and shortly after the 2008 large outbreak of HFMD were evaluated by the microneutralization test. The geometric mean titer (GMT) was calculated and compared. Statistical significance was taken as P < 0.05.

**Results:**

The seroprevalence data showed a continuous circulation of EV71 and CA16 in Guangdong province China in 2007–2009. The low positive rate in 2009 correlated well with the unprecedented outbreak of HFMD in 2010. Age related increase of seroprevalence was identified in 1–3 years old children for EV71 and in 1–5 years old children for CA16 in Guangdong province. High GMT of EV71 and CA16 antibody titers were also found for these age groups.

**Conclusions:**

All of the above findings indicated common infections for these age groups. And they should clearly be at the top of the priority in periodical seroprevalence survey and future vaccination campaign.

## Background

Hand, foot and mouth disease (HFMD) is a common pediatric illness [1]. It is characterized by 3–4 days of fever and then the development of vesicles on the palmar and plantar skin, buccal mucosa and tongue [2-4]. This illness as itself is mild and self-limited [5]. While in some cases, accompanied with other neurological complications, HFMD may also lead to severe outcomes and even death [6-8]. Children under 10 years old especially those less than 5 are the most susceptible population for reasons that are not clearly understood [2,9-11].

The first viral agent identified for HFMD is Coxsackievirus A 16 (CA16) [12]. Belonging to the picornaviridae family, this virus, associated with Enterovirus 71 (EV71), is responsible for nearly all of the HFMD epidemics in Southeast Asia [13,14]. In Japan, CA16 was reported to be a prevalent type, while EV71 recurrent every 3 years [10,15]. In Singapore, from 2001 to 2008, the predominant EV strains isolated from HFMD cases were CA16 and EV71 [16,17]. In China, EV71 and CA 16 are also frequently reported to co-circulate and cause HFMD, although the major etiologic agent identified from hospitalized cases was EV71 [18,19].

Many large HFMD outbreaks with severe and fatal outcomes in Southeast Asia have recently been described [4,20]. In Taiwan, the most severe HFMD outbreak occurred in 1998, resulting in 405 severe neurological cases and 78 deaths [21,22]. In China, the recent outbreaks of HFMD were initiated from 2007 in Shandong province. Then a national widespread epidemic occurred in 2008, with more than 176,000 reported human cases [23]. Contrary to HFMD outbreaks in other Asian countries, the epidemics in China were more lasting [24]. Unprecedented HFMD outbreaks occurred in the following years. By the end of 2010, a total of 3,419,149 cases and 1384 fatal cases were reported [25,26]. It is said that the recently occurred HFMD epidemics have become a serious public health problem in Southeast Asia, especially in China.

Currently,only a few studies have been conducted to investigate the human immunity to HFMD in China [27,28]. The seroepidemiology of EV71 infection before, during and shortly after the epidemics could pave ways for prophylactic intervention strategies. In addition, several EV71 vaccine candidates are being developed in mainland China from 2008 [29-31]. And some of them are at various stages of clinical development [32,33]. To better establishing the immunization program against EV71 and CA16 infection, seroepidemiological surveillance is urgently needed. In this study, we conducted a cohort study in Guangdong province, China. Children less than 10 years old were enrolled. The levels of EV71 and CA16 specific antibodies in children aged between 1 and 9 years were evaluated and compared for 3 years (2007–2009).

## Methods

### Serum samples collection

The material used in this study is stored serum samples collected from the health children ≤9 years of age who had participated in seasonal immune status surveillance at Guangdong Provincial Centre for Disease Control and Prevention, China, from 2007 to 2009. Survey questionnaire was completed by trained interviewers and included information on the subject’s age, gender, vaccination history (over the past year) and presence/absence of illnesses (over the past year). All children had no sign of disease at the time of sample collection. The serum samples were stored at −80°C until testing. For the use of these serum samples, written informed consents from all participants (their parents or legal guardians) involved in survey were obtained. Serum samples from children who reported fever and vesicular exanthema on their hands, feet, mouths, or buttocks (distinct clinical presentation of HFMD) over the past year were excluded in this study and were classified into seven age groups (1, 2, 3, 4, 5, 6–7 and 8–9 years). Each group has 35–40 samples, except age group 1, 2 years in 2007(3 in 1 year group, 24 in 2 year group) and 8–9 years group in 2008 and 2009 (24 and 23 samples respectively). The sex ratios of boys to girls were 1.36:1, 1.43:1, and 1.25:1 respectively. The demographic profile of the subjects is shown in Table 1. The study was approved by the ethics committee of the Guangdong Provincial Center for Disease Control and Prevention, and was in compliance with the Helsinki Declaration.

**Table 1:** Demographic profile of the subjects in three years studied

	**2007**	**2008**	**2009**
	**No. samples**	**No. samples**	**No. samples**
**Age groups (years)**			
1	3	35	36
2	24	34	35
3	36	39	38
4	40	38	37
5	39	39	39
6-7	40	39	37
8-9	40	24	23
**Gender**			
Male	128	146	136
Female	94	102	109
Total	222	248	245

### Neutralizing antibody assay

The neutralizing antibody (NA) tests were performed as previously described [34]. To define the EV71 neutralizing antibody, EV71/Guangdong/EV039/2009 (C4 genotype) strain was used, which is isolated from a fatal case in 2009. For CA16, CA16 Guangdong/CA010/2009 (A genotype) strain was used. All of the neutralizing antibody assays were run in 96-well microplates. Serum samples were inactivated at 56°C for 30 minutes before use, diluted two-fold from 1:8 to 1:1,024, and then incubated at 37°C for 2 hours with equal volumes of 100 half tissue culture infective doses (100 TCID50) of EV71 or CA16. After the incubation period, 1 × 10^5^ cells /ml rhabdomyosarcoma cell lines (RD) were added to each well. Finally, these plates were incubated in a 5% CO2 incubator at 37°C for 7 days. And the CPE was observed with an inverted microscope from the fourth day. All the diluted samples were tested in duplicate. Cell control, serum control and virus control were included in each plate. Viral back titration was conducted in each test. The antibody titer of the sample was defined as the highest dilution that could inhibit cytopathic effect (CPE) development in 50% of the virus-infected wells. A titer equal to or greater than 8 were considered as seropositive.

### Statistical analysis

Statistical analyses were performed with SPSS version 13.0. Differences with an error probability of P < 0.05 were regarded as significant. For categorical data, we used Chi-square testing and Fisher Exact testing as appropriate. Neutralizing antibody titers of positive serum samples were log-transformed to calculate the geometric mean titer (GMT) and 95% confidence intervals (CI). Kruskal–Wallis test was used for comparison of the GMT by year and age group. Antibody titers >1:1204 were assigned a value of 1204.

## Results

### Overall seroprevalence of EV71 and/or CA16

EV71 and CA16 NA are widely identified in the population in three years tested (Table 2). The seroprevalence of NA to EV71 was found to be 44.6% (95% CI 38.0–51.0%), 46.4% (95% CI 40.2–52.6%) and 31.8% (95% CI 26.0–37.6%) in 2007, 2008 and 2009 respectively. EV71 NA seroprevalence in 2009 showed an obvious decreased trend when compared to that of 2007 and 2008 (p < 0.05). Related to EV71, higher seroprevalence was observed for CA16 NA in same years except for 2008 (p < 0.05). In 2007, 70.3% (95% CI 64.3–76.3%) individuals were seropositive for CA16 NA. Seroprevalence in 2008 and 2009 was significantly lower than that of 2007 (p < 0.05), with similar values ranged between 51.6% (95% CI 45.4–57.8%) and 47.3% (95% CI 41.0–53.6%).

**Table 2:** Overall seroprevalence of EV71 and/or CA16 antibody

		**No. (%) positive**		
**Year**	**No. tested**	**Enterovirus 71**	**Coxsackie A16**	**Co-infection**
**2007**				
Male	128	52(40.6)	92(71.9)	39(30.4)
Female	94	47(50.0)	64(68.1)	33(35.1)
Total	222	99(44.6)	156(70.3)	72(32.4)
**2008**				
Male	146	64(43.8)	77(52.7)	43(29.5)
Female	102	51(50.0)	51(50.0)	30(29.4)
Total	248	115(46.4)	128(51.6)	73(29.4)
**2009**				
Male	136	47(34.6)	68(50.0)	31(22.8)
Female	109	31(28.4))	48(44.0)	19(17.4)
Total	245	78(31.8)	116(47.3)	50(20.4)

Of all tested individuals, 32.4% (95% CI 25.8–38.2%) and 29.4% (95% CI 23.3–34.7%) revealed neutralizing antibodies against both viruses in 2007 and 2008 (Table 2). Then the seroprevalence decreased greatly (p < 0.05), showing a value of 20.4% (95% CI 15.0–25.0%) in 2009.

No significant gender specific difference in seroprevalence was observed for both EV71 and CA16 NA (Table 2). 40.6% (95% CI: 32. 1 – 49. 4%) of boys tested EV71 NA seropositive, compared to 50.0% (95% CI: 40.0 – 60.2%) among girls in 2007. While for CA16 NA, the seroprevalence was 71.9% (95% CI: 64.0–79.6%) and 68.1% (95% CI: 58.7–78.9%) respectively. Values in 2008 were 43.8% (boys, 95% CI: 36.2–52.4%) and 50.0% (girls, 95% CI: 40.5–60.3%) for EV71 NA, 52.7% (boys, 95% CI: 45.2–61.2%) and 50.0% (girls, 95% CI: 40.5–60.3%) for CA16 NA. 34. 6% (95% CI 26.4–43.5%) boys and 50.0% (95% CI 41.2–59.3%) boys showed neutralizing antibodies to EV71 and CA16 respectively in 2009. The proportion of EV71 /CA16 seropositive girls was 28.4%/44.0%.

### Age-dependent seroprevalence of EV71 and/or CA16

**Figure 1:**
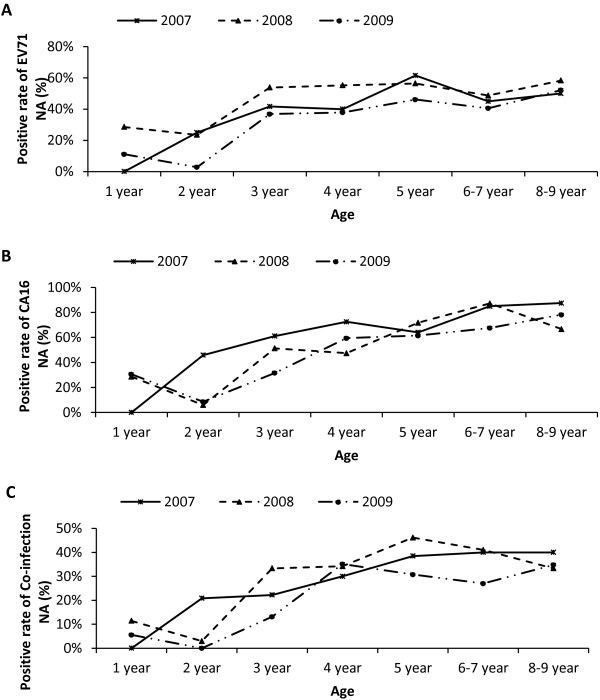
**Age-dependent seroprevalence of EV71 and/or CA16 antibody.** Age-related seroprevalence of neutralizing antibodies to EV71 **(A)**, CA16 **(B)**, or both of these two viruses **(C)** in individuals in Guangdong, China.

The analysis of the different age groups (n = 35–40) revealed a general increasing trend for EV71 NA seroprevalence in all of the three years tested, except for a slight dip at the age of 2 years group in 2008 and 2009 (Figure 1A). The percentage of EV71 NA positive individual increased from 0%, 28.6% and 11.1% in the first group (age 1 year group; 2007, 2008 and 2009 respectively) to plateau values no less than 40%, 48.7% and 36.8% among 3 to 9 years old individual. No obvious rise or decline was observed for these age groups.

While for CA16, it showed much higher seroprevalence than EV71 in nearly all of the age group in three years tested except in year 2008. Similar with EV71 NA seroprevalence, an age related increase of CA16 NA seropositive individuals was observed (Figure 1B). In 2007, children 2 years old had a seroprevalence of 45.8%, and thereafter reached to at least 61.1% in 3–5 years groups, then this value stated at approximately 85% for those aged 6 or older. Compared to 2007, the age dependent curves in 2008 and 2009 both showed significant reduction at 2 years group. CA16 NA seroprevalence declined from 28.6% and 30.5% at 1 year group to 5.8% and 8.5% at 2 year group in 2008 and 2009 respectively. Then these values steady increased to at least 66.7% for children aged 5 or old in 2008, and to 59.5% for children aged 4 or old in 2009.

A moderate percentage was obtained when testing neutralizing antibodies against both EV71 and CA 16 (Figure 1C). Children among 2–3 years old had a value of 20.0%, and 30.0% at age 4, and then reached at 40.0% for the subsequent groups in 2007. In 2008 and 2009, the lowest level of NA seroprevalence for these two viruses was 2.9% and 0.0% respectively, both were found at age 2. Thereafter the NA seroprevalence stated at appoximately 30.0% among 3–9 years children.

### Geometric mean titer distribution of EV71 and CA16 neutralizing antibodies in seropositive individuals

**Figure 2:**
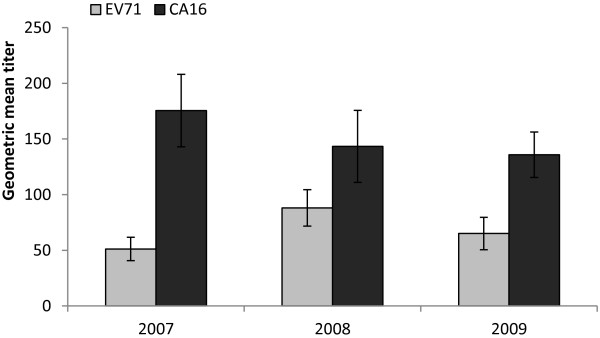
**Geometric mean titer (GMT) of EV71 and CA16 antibody in seropositive individuals.** The lines indicate 95% confidence interval.

To analyze the immunity level, the geometric mean titer (GMT) of EV71 and CA16 neutralizing antibodies in seropositive individuals was tested. The overall EV71 NA showed a moderate level, with GMT values of 51.6 (95% CI:40.6-64.5), 88.1 (95% CI:71.3-108.9) and 56.6 (95% CI:41.6-76.8) in 2007, 2008 and 2009 respectively (Figure 2). For CA16, significant higher values were observed than those for EV71 in each year tested (p < 0.05) (Figure 2). The highest CA16 antibody level was identified in 2007, with a value of 175.5. Then the GMT values gradually declined to 143.4 and 135.8 in 2008 and 2009 respectively (Figure 2).

**Figure 3:**
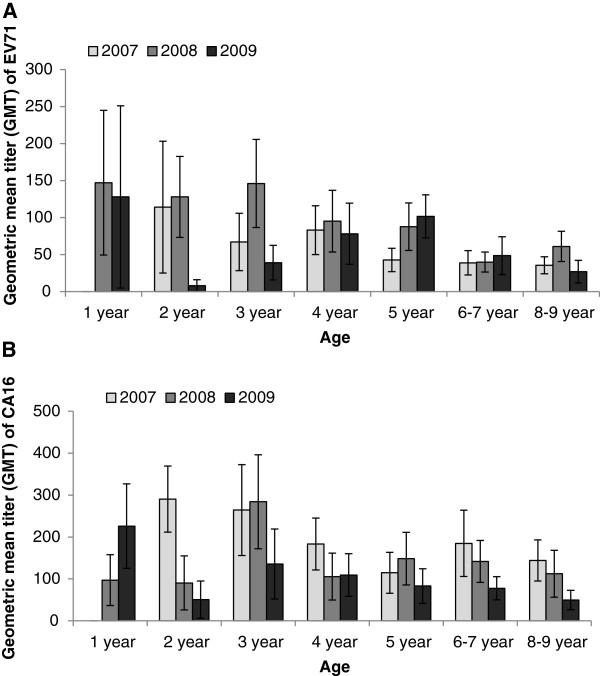
**Geometric mean titer (GMT) of EV71 and CA16 antibody by age group.** Age-related geometric mean titer (GMT) of EV71 **(A)** and CA16 **(B)** antibody in seropositive individuals. The lines indicate 95% confidence interval.

GMT values of EV71 NA were slightly higher in 1–5 years group than in those 5 years older. The highest GMT of EV71 in 2007 was in children aged 2 years group (GMT 114.0). Then this value reduced across age groups, fell to approximately 40.0 among those 5 years or older (Figure 3A). Compared to 2007, the GMT in 2008 was relatively high in most of the age groups. Children in 1–5 years groups had values no less than 87.7. Then the immunity level decreased to 39.8 and 60.9 in children aged 6–7 and 8–9. In 2009, the highest GMT was identified in 1 year old children (GMT 128.0). Contrary to 2007, the 2 years group had a lowest value of 8 in 2009. Then this value gently increased with age, rising from 39.0 to 101.6 in 3–5 year groups. Again, for those aged more than 5 years, the GMT in seropositive individuals was relatively low. No one was found to be more than 50.0. For CA16 (Figure 3B), the GMT values were approximately equally distributed in 2007 and 2008. Significant higher values were only observed in 2–3 years groups in 2007 and in 3 year group in 2008. While in 2009, declining trend across age was identified, with a sudden dip at age of 2 years.

## Discussion

In humans, humoral immunity with neutralizing antibodies played central roles in anti viral infection [35-37]. Recent vaccine studies in animal model also demonstrated the protective effects of neutralizing antibody in EV71 challenge [38,39]. In addition, surveillance data in other countries also suggested a causative role for the decreased herd immunity in susceptible population during HFMD outbreaks [40]. To date, few surveillance data were reported on seroepidemiology of EV71 and CA16 in China. In this study, the neutralizing antibodies against EV71 and CA16 among children in Guangdong were investigated. Seroprevalence changes before, during and shortly after the national widespread epidemic in 2008 were described.

In China, HFMD was first reported in Shanghai in 1981, and since then, sporadic cases of HFMD in other provinces including Beijing, Fujian, Shandong, Hubei, Jilin were reported in 1980s. Recently the epidemic situation of HFMD in China tended to be more serious. In 2007, nearly 40,000 HFMD cases were reported in Shandong and over 10,000 cases were recorded in large cities such as Beijing and Shanghai [41]. However, little reports have described the HFMD situations in Guangdong in 2007. Our results suggested moderate EV71 infections occurred among the children in Guangdong before the large HFMD epidemic in 2008. The overall seroprevalence of EV71 NA in 2007 was similar to those in 2005 in Guangdong, as well as those in 2006–2007 in Lu’an [42,43]. About half of children less than 10 years old had no neutralizing antibody against EV71. Compared to EV71, significant higher positive rate of neutralizing antibody against CA16 in 2007 was identified. This was contrary to a previous study, which suggested more common EV71 infections in South China in 2005 [42]. The high CA16 seroprevalence in 2007 indicated that frequent asymptomatic and/or unrecognized CA16 infections have occurred before and in this year. In 2008, among 936 laboratory-confirmed cases, EV71 was found in 59% and CA16 in 26% [19]. Considering the protective effects of neutralizing antibody in viral infection, the epidemiology surveillance data in 2008, to some degree, had verified our results. The seroprevalence of EV71 and CA16 in this year could serve as HFMD trends predictors for next year.

In March 2008, a serious HFMD epidemic with 20 deaths broke out in Fuyang City, Anhui Province, and subsequently spread quickly in almost all provinces of China [44]. After that, the Ministry of Health of China classified HFMD as one of the category “C” notifiable diseases. According to the Guangdong HFMD web-based surveillance system, a total of 47,660 HFMD cases were reported in 2008. And this number almost doubled in 2009 (92,998 reported cases), was five-fold in 2010 (230,978 reported cases) [41]. To correlate the level of immune protection to the incidence rate of HFMD cases, seroprevalence of EV71 and CA16 NA in and after the 2008 were also investigated. We didn’t find significant higher positive rate of EV71 and CA16 in and after 2008. Previous study suggested that HFMD presents a seasonal pattern every 2–3 years [45]. Together with the increased incident rates of HFMD cases in 2009–2010, our results suggested that the peak of recent HFMD epidemic cycle in Guangdong was not in year 2008 but in the years 2009 and 2010. Consistent with these, Yu, et al. identified that children in Lu’an had significant higher seropositive rate in 2010 when compared to that before 2008 [43]. The ensuing HFMD epidemics from 2008 to 2010 largely increased the exposed chance of viral infection and thus the seropositive rate of viral NA in children.

EV71 and CA16 were highly diverse in the nucleotide sequences of structure proteins which serve as major antigen in host immune response. Li et al. used VP1s and VP4s as antigens to detect of serum antibodies against EV71 and CA16 [46]. Their results suggested immunological reaction to VP1 and VP4 of both EV71 and CA16 was different. The study from the clinical patient also indicated that individuals with or without prior EV71neutralizing antibody showed a similar incidence of non-71 Enterovirus infection [47]. EV71 and CA16 were the most common causes of HFMD diseases in recent epidemics in China [44]. To evaluate the immune protection level against HFMD, we also calculated the proportion of individuals that were positive for both the EV71 and CA16. In total, about 30.0% of all tested individuals had antibodies both to EV71 and to CA16 before and during the 2008 epidemic, while this rate greatly reduced to 20.0% in year 2009. The large number of susceptible individuals may be partly responsible for the large outbreak of HFMD in Guangdong in 2010. Similar with reports in other countries, the dates in our study also proved that individual susceptible probability depends on the protective level of neutralizing antibodies. Epidemic occurred when herd immunity decreased.

As a common febrile illness of early childhood, HFMD occurred mainly among children ≤5 years old. In Guangdong, a total of 47,660 and 92,998 cases have been reported to the provincial surveillance system in 2008 and 2009 respectively. The number of children ≤5 years old accounted for the largest proportion (from 87.5% to 93.3%) [48]. These were highly consistent with the age related seroprevalence trends in our study. In our results, both EV71 and CA16 NA rise with age among children less than 5 years and reach a plateau thereafter [34,49]. We also observed significant reduction at 2 years group, the most susceptible population that had the highest incidence rates of HFMD in 2008 and 2009 epidemic year in Guangdong. In 2007, the lowest seroprevalence was identified in 1 year old children. While for children in 2 year old group, slightly high positive rate was shown. Epidemic waves before and in this year could not be traced. Whether this different seroprevalence trend in 2007 provided an accurate picture, or it just was the result of very small number of cases in 1 year group remains to be determined by future investigations.

The GMT for EV71 and CA16 was also analyzed. All the seropositive individuals in three years tested showed relative high CA16 NA levels (Figure 2). Unlike a previous report conducted in Jiangsu province China, which showed low antibody titers against CA16 in children aged 2–15 years, the high GMT observed in this study suggested common CA16 infections in Guangdong province [27]. In addition, our data also suggested that most of the EV71 infections are acquired when they were less than 5 years old, while for CA16, mainly occurred among 1–3 years groups (Figure 3), because the highest antibody titers were observed in these age groups and neutralizing antibody titers were high at early stage of infection. GMT for both the EV71 and CA16 were all declined among children 5 years or older, which account for about 10% of the total reported HFMD cases [48]. The relative low level of EV71 and CA16 NA in these less susceptible populations indicated a critical role for the long-lasting immunity rather than immunity level in protecting against viral infection.

## Conclusions

Altogether, the seroprevalence data in our study showed a continuous circulation of EV71 and CA16 in Guangdong province China in 2007–2009. The relative high seropositive rates in healthy children indicated many clinically silent EV71/CA16 infections in these years. These seropositive children that had asymptomatic or unrecognized EV71/CA16 infection may serve as a reservoir for continued viral spread in the population, and should be taken into account when the government develops and implements public health interventions. Consistent with previous studies, age related increase of seroprevalence was also identified in 1–5 years children in Guangdong, which indicated common infections for these age groups. And they should clearly be at the top of the priority in periodical seroprevalence survey and future vaccination campaign.

## Competing interests

The authors declare that they have no competing interests.

## Authors’ contributions

YL and KC drafted the manuscript. LW, SJ, LJ, ZH, GD, MC, ZW and XH carried out the laboratory tests, LH, ZY and LJ performed the statistical analyses. All authors read and approved the final manuscript.

## Pre-publication history

The pre-publication history for this paper can be accessed here:

http://www.biomedcentral.com/1471-2334/13/322/prepub
